# The Impacts of Different Biological Treatments on the Transformation of Explosives Waste Contaminated Sludge

**DOI:** 10.3390/molecules26164814

**Published:** 2021-08-09

**Authors:** Arturo Aburto-Medina, Esmaeil Shahsavari, Mohamed Taha, Andrew Bates, Leon Van Ieperen, Andrew S. Ball

**Affiliations:** 1 School of Science, RMIT University, Bundoora, VIC 3083, Australia; e.shahsavari@gmail.com (E.S.); moohamedtaha@yahoo.com (M.T.); andy.ball@rmit.edu.au (A.S.B.); 2 Department of Biochemistry, Faculty of Agriculture, Benha University, Moshtohor, Toukh 13736, Egypt; 3 Australian Munitions, Bayly Street, Mulwala, NSW 2647, Australia; andrew.bates@australianmunitions.com (A.B.); Leonvanleperen@australianmunitions.com (L.V.I.)

**Keywords:** dinitrotoluene isomers, bioaugmentation, *Burkholderia*, qPCR, Illumina, 16S rDNA

## Abstract

The dinitrotoluene isomers 2,4 and 2,6-dinitrotoluene (DNT) represent highly toxic, mutagenic, and carcinogenic compounds used in explosive manufacturing and in commercial production of polyurethane foam. Bioremediation, the use of microbes to degrade residual DNT in industry wastewaters, represents a promising, low cost and environmentally friendly alternative technology to landfilling. In the present study, the effect of different bioremediation strategies on the degradation of DNT in a microcosm-based study was evaluated. Biostimulation of the indigenous microbial community with sulphur phosphate (2.3 g/kg sludge) enhanced DNT transformation (82% transformation, from 300 g/L at Day 0 to 55 g/L in week 6) compared to natural attenuation over the same period at 25 °C. The indigenous microbial activity was found to be capable of transforming the contaminant, with around 70% transformation of DNT occurring over the microcosm study. 16S rDNA sequence analysis revealed that while the original bacterial community was dominated by Gammaproteobacteria (30%), the addition of sulphur phosphate significantly increased the abundance of Betaproteobacteria by the end of the biostimulation treatment, with the bacterial community dominated by *Burkholderia* (46%) followed by *Rhodanobacter*, *Acidovorax* and *Pseudomonas*. In summary, the results suggest biostimulation as a treatment choice for the remediation of dinitrotoluenes and explosives waste.

## 1. Introduction

Explosives are a global term that refers to specific energetic materials containing energy stored (e.g., trinitrotoluene, TNT) in one form or another (chemical, nuclear or pressurised gas) that can readily be exploded. Explosive materials are required for a wide range of applications, including mining, construction, quarrying, space exploration and rockets, missiles and gun propellants [1]. As a result, a huge amount of explosive waste is produced globally. Currently, 2,4 and 2,6 DNT are listed as the major explosive contaminants of military sites [2], with extensive contamination of both soil and groundwater. In Germany, 2340 sites are listed as contaminated with residues of explosives [2]. Nitroaromatic compounds, such as TNT, are recalcitrant and persist in the environment (as a liquid or solid) for years; aerobic biodegradation is prevented due to the compounds electron-withdrawing characteristic resulting from the presence of multiple nitro groups (-NO_2_). Mononitrotoluenes are more readily biodegradable than TNT and can be degraded by bacteria without prior adaptation [3]. Explosive materials (such as TNT and DNTs) can cause adverse health, ecotoxicological, cytotoxic, teratogenic and carcinogenic effects on a wide range of ecological receptors including microorganisms, algae, invertebrates, animals, plants, vertebrates and humans and therefore pose serious threats to the entire environment [4]. The occurrence of both dinitrotoluene isomers 2,4 and 2,6-DNT in groundwater and soils is a particularly widespread problem [5,6,7,8].

Explosive contaminants can enter living organisms by skin contact, inhalation or consumption of contaminated vegetation or water and consequently can damage living organisms [9].

Thus, the removal of these compounds from the environment is paramount. Traditional removal treatments, such as open detonation and burning, have been widely applied for the decontamination of ammunition wastes; however, these techniques are environmentally unfriendly, labour intensive and prone to secondary contamination. Other removal and disposal strategies focus on dig and dump approaches; this is both expensive and unsustainable. In contrast, the application of a biological treatment using microbial consortia capable of mineralising DNT, resulting in harmless end products (microbial biomass, H_2_ and CO_2_) represents a sustainable remediation approach. Moreover, these contaminants have been reported as biodegradable in both aerobic [3,10,11,12,13] and anaerobic conditions [14,15,16,17,18]. Indeed, pure cultures capable of 2,4-DNT degradation have recently been isolated; they include an *Arthrobacter* strain isolated from crude oil-contaminated soil [11], *Rhodococcus pyridinovorans* NT2 [12] and *Shewanella marisflavi* EP1 [16], which operates in anaerobic conditions. However, despite the intensive work conducted on the mineralisation of DNTs since the report of the first microbial strain showing complete degradative capability in 1991 [19], the highest catabolisation efficiency remains less than 50% [3]. A consortium named UHasselt Sofie 3 (UHS3), formed by *Burkholderia* HC114, *Variovorax paradoxus* VM685, *Bacillus*, *Pseudomonas mandelii* HC88 and *Ralstonia* HC90 was successfully employed for the degradation of 2,4-DNT and recorded faster degradation rates than other test consortia [4].

While some microorganisms perform the degradation of DNT in aerobic conditions, the marine strain *Shewanella marisflavi* is capable of the anaerobic degradation of DNT [16]. The *S. marisflavi* strain EP1 was found to be capable of the complete mineralisation of 2,4-DNT over a range of environmental conditions (pH 7–9, 4–40 °C and 2–8% NaCl). We recently published a review of the microorganisms involved in the degradation of the DNT isomers [20]. Our literature survey confirmed that most of the reports have focused on pure DNT degradation and microbial isolation; little attention has been paid to the bioremediation of DNT in explosive waste. While many recent studies have demonstrated the capability of different microorganisms (bacteria and fungi; pure strains and/or defined mixed culture) to adapt and utilise high concentrations of DNT as the sole carbon, nitrogen and energy source (such as *Burkholderia cepacia* and *Hydrogenophaga palleronii*), to date, there has been no development of a commercial bioremediation technology for DNT [1].

Our previous research evaluated bioremediation approaches for the degradation of explosive waste. The results confirmed that bioremediation can be applied to remediate TNT, cyclonite (RDX) and other explosive chemicals [21]. That study involved the use of microcosm studies to successfully remediate soil spiked with TNT chips. We found that using previously bioremediated hydrocarbon contaminated soil resulted in a 70% increase in the remediation of TNT compared with the control [21]. Effective bioremediation strategies remain a current area of interest, not only to improve and accelerate microorganisms’ performance but also to address the environmental requirement to maximise mineralisation and removal of explosive pollutants from contaminated soil, which is paramount. This study aimed to assess and enhance the transformation potential of an indigenous community chronically exposed to DNT and, in turn, provide a sustainable biological treatment for the remediation of DNT-contaminated sludge.

## 2. Materials and Methods

### 2.1. Mesocosms

DNT-contaminated sludge (10 kg) was obtained from Australian Munitions. The DNT level in the sludge was 30% (300 g/L). The properties of DNT-contaminated sludge are shown in Table 1.

**Figure 1:**
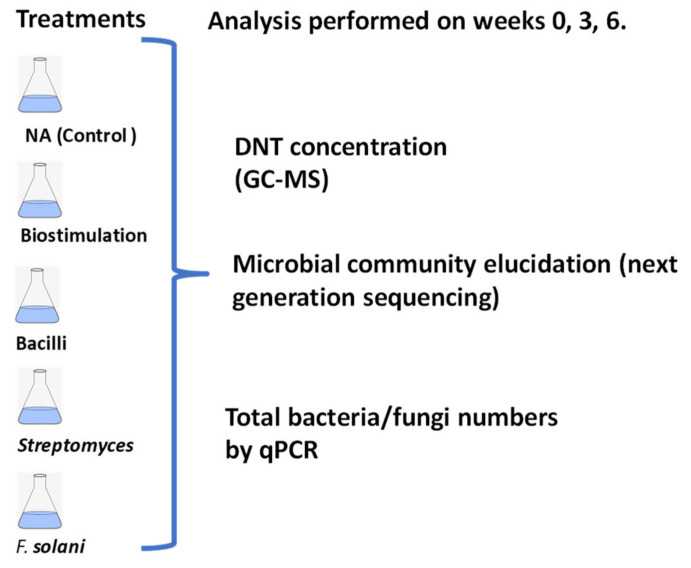
Scheme of the methodology followed in this study. NA: Natural Attenuation; five treatments were set up and analysed at the start of the experiment, weeks 3 and 6.

A total of 5 treatments were assessed (Table 2, Figure 1), including three bioremediation approaches. Three replicates were used for each treatment. Mesocosms were prepared by adding 250 g of pond sludge, amended where necessary (biostimulation and augmentation treatments) by the addition of sulphur phosphate (added at 2.3 g/kg sludge); based on the nutrient status of the waste). All mesocosms were incubated at 25 °C for up to 6 weeks, with shaking (150 rpm).

For biostimulation, the C:N:P ratio was balanced to provide optimal conditions for biodegradation of DNT in sludge. This involved the addition of sulphur phosphate amendment to achieve a 100:10:1 molar ratio of C:N:P (Redfield Ratio) [22,23]. The contaminant DNT contained both C and N (C_7_H_6_N_2_O_4_). The addition was dependent upon the elemental composition of the waste stream (provided by Australian Munitions) determined prior to the commencement of the study.

Bioaugmentation: A consortium composed of 10 strains or isolates, including bacteria and fungi, were used as the bioaugmentation treatment (Table 2).

The bacilli consortium was selected due to its availability in our laboratory. *Bacillus* strains have previously been shown to be involved in the degradation of at least one of the DNT isomers [3,4,24] and to degrade other environmental pollutants. For example, the *Bacillus* consortium was successfully used for the biodegradation of phenol in wastewater [25], hydrocarbon in soil [26] and groundwater [27]. Weekly sampling starting prior to the incubation occurred over 6 weeks. Samples were stored at −20 °C prior to analyses as described below.

### 2.2. DNT Concentration

Aliquots from each treatment were analysed by Australian Munitions, (Mulwala, Australia) to determine the concentration of DNT isomers using gas chromatography–mass spectrometry, as described previously [28], at the beginning of the experiment, Week 3 and Week 6.

### 2.3. Microbial Community Analysis (16S DNA Illumina MiSeq Sequencing)

Total genomic DNA was extracted from 0.25 g of sludge using a MoBio Power Soil DNA extraction kit, according to the manufacturer’s instructions (MoBio Laboratories Inc, Carlsbad, CA, USA). The metagenomic library was prepared using an Illumina Nextera^®^ XT Index Kit (Illumina, San Diego, CA, USA), following the manufacturer’s instructions. DNA from the library was quantified using a Qubit^®^ 2.0 Fluorometer (Life Technologies, Carlsbad, CA) and 2100 Bioanalyzer (Agilent Technologies, Santa Clara, CA, USA). Samples were then pooled, and libraries mixed with Illumina-generated PhiX control libraries and our own genomic libraries and denatured using fresh NaOH and run on a MiSeq platform (Illumina, San Diego, CA, USA).

### 2.4. Quantification of Total Fungi, Bacteria (qPCR)

Quantification of 16S rDNA was performed using quantitative polymerase chain reaction (qPCR). qPCR reactions (25 μL volume) were performed in a Rotor-Gene Q series (Qiagen). Each reaction contained the Kapa Sybr Fast mastermix (10 μL, KAPA), RNase-free sterile water (8.2 μL), forward primer 341F (0.4 μL, 10 pmol/μL), reverse primer 518R (0.4 μL) and DNA template (1 μL). RT-qPCR amplification conditions were performed as described previously [29]. Universal primers 341F and 518R [30] were used to amplify 16S rRNA genes.

For quantification of total fungi, ITS region genes were amplified using ITS1F and 5.8S primers [31]. Two negative controls (containing water instead of template DNA) and two positive control samples were included in each qPCR run. Briefly, qPCR was performed in 20 μL reaction volumes containing 10 μL of 2X Kapa SYBR Fast qPCR Master Mix (Kapa Biosystems), 0.4 μL of each primer (10 pmol/μL), 8.2 μL of molecular-biology-grade water and 1 μL of template DNA or distilled water (negative control). Amplifications were carried out with the following temperature profiles: an initial denaturation step at 95 °C (5 min) followed by 40 cycles of 95 °C denaturation (10 s), annealing temperature 55 °C for 16S rDNA and 53 °C for ITS region genes (30 s) 72 °C extension (30 s), 80 °C primer dimer removal and signal acquisition (10 s) [31,32]. 16S rDNA gene copy numbers were calculated by relating the treatments’ CT value to a standard curve. The CT values from standard dilutions were plotted against the log of their initial copy number followed by the generation of a standard curve using linear regression. The gene copy numbers were expressed as log_10_ of gene copy numbers per g dry sludge.

### 2.5. Data Analysis

One-way analysis of variances (ANOVA) was performed for multiple data using IBM SPSS software (version 22) to check for quantitative differences between samples. Data were significantly different at *p* = 0.05.

Sequencing data obtained from metagenomic analysis were analysed using the 16S Metagenomics App in Illumina Basespace (San Diego, California). Sequences were quality trimmed, filtered and processed using the Quantitative Insights Into Microbial Ecology (QIIME) package available from the Illumina Basespace website [33]. The obtained sequencing data from the QIIME were further analysed by MEGAN 6 [34].

An Operational Taxonomic Units (OTU) genus-level table was imported into Primer 7 software; the data were standardised and transformed using square root. Shannon diversity and richness indices were also calculated using Primer 7 software, as described in the software manual. The standardised and transformed data were subjected to resemblance analysis using S17 Bray Curtis similarity resemblance measure and cluster analysis applied to generate plot dendrograms using the group average cluster mode. In addition, principal coordinate analysis (PCoA) was performed using Primer 7 [35].

## 3. Results and Discussion

### 3.1. DNT Remediation

**Figure 2:**
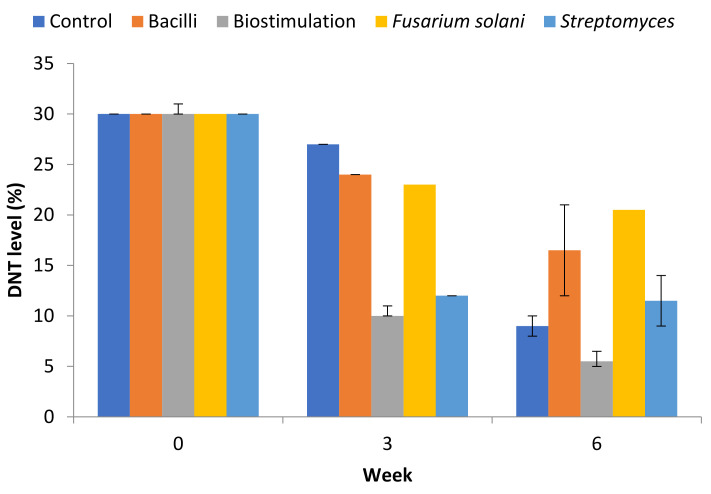
Concentration of DNT over 6 weeks at 20 °C. Results shown represent the mean of duplicates, with standard errors shown.

The sludge was homogenised by mixing the samples prior to starting the experiment. Flakes of DNT were visible and evenly distributed in the sludge. The concentration of DNT in the sludge on Day 0 was 30% (300 g/L), with 2,4 DNP representing almost 100% of the DNT present. DNT concentrations during the incubation are presented in Figure 1. The results indicated that all treatments, including the control, resulted in the transformation of DNT relative to Day 0 during the 6-week mesocosm incubation. This represents an important finding as it suggests that the indigenous microbial community was capable of transforming the contaminant. However, biostimulation (sulphur phosphate added at 2.3 g/kg sludge) and bioaugmentation (the addition of *Streptomyces* sp.) treatments showed the greatest transformation rate with an average of 66 and 60%, respectively, at Week 3 relative to the control, which showed an average of 10% reduction. However, the control sludge showed a significant reduction in DNT levels at Week 6, with the level of DNT dropping from 270 g/L to 90 g/L (70% reduction rate). Overall, the presence of nutrients (biostimulation treatment) showed the maximum effect as the level of DNT decreased to 5.5% (55 g/L, 82% reduction rate). The results showed that the addition of either the Bacilli or fungus were not as efficient when compared to natural attenuation and biostimulation treatments by Week 6; the DNT concentration decreased from 300 g/L to 165 g/L and 205 g/L for Bacilli and fungal treatments, respectively (Figure 2).

The results showed that the original sludge contained DNT-degrading microbes. However, it seems the physicochemical conditions of the original sludge, such as the lack of oxygen and nutrients (such as P), prevented the activity of those microbes. Therefore, the addition of P with shaking, led to the activation of the degrading microbial communities involved in the transformation of DNT. Furthermore, the addition of P might enhance the transformation of DNT due to increasing key-microbial abundance, as previously reported [3]. Although natural attenuation is considered the simplest and cheapest biodegradation strategy, the effectiveness of the indigenous microorganisms is limited.

### 3.2. Total Microbial Community Analysis Quantitative PCR

**Figure 3:**
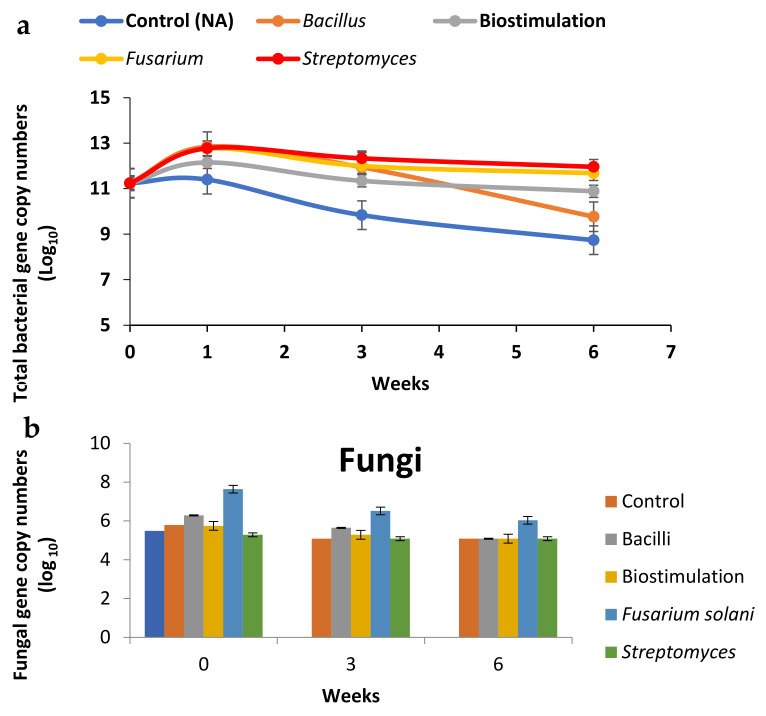
(**a**) Total bacteria and (**b**) fungi gene copy abundance over 6 weeks in different treatments. Results are the means of triplicates with standard errors shown.

To understand the influence of bioremediation treatments on the microbial ecology of the waste stream, total bacterial numbers (log_10_ scale) were monitored in the microcosm study based on quantifying gene copy numbers (Figure 2). The total number of bacterial 16S rDNA gene copies was 11.2 log_10_ CFU/g dry sludge on Day 0. The highest copy number (12.8 log_10_ /g dry soil) was detected in the biostimulation treatment at Week 1; the lowest was observed in the control at Week 6 with an average of 8.7 log_10_ /g dry sludge. The results indicated that total bacterial numbers increased in each of the treatments compared to the control; for the bioaugmentation treatment, this resulted in a 40-fold increase. Interestingly, this was also observed in the biostimulation treatment, even though no additional bacteria were included, confirming that the additional nutrients led to an increase in the indigenous microbial biomass (Figure 3).

As expected, the largest numbers of bacteria were observed in all the microorganism-amended treatments (Bacilli, *Fusarium solani* and *Streptomyces* treatments) during the initial 7-day incubation following addition. Although the total numbers of bacteria decreased throughout the incubation (Weeks 3 and 6) for all the treatments, they were still higher than the bacterial numbers in the control (natural attenuation) microcosm. Interestingly, by Week 6, the number of bacteria present in the treatment bioaugmented with the *Bacilli* consortium was significantly reduced, suggesting their unsuitability for this environment. Regarding total ITS gene copy (reflecting total fungi), the results showed that a slight decrease in the number of gene copies was observed in all treatments during the time. There were no significant differences among treatments except *Fusarium solani* (FS) at Week 6. The highest numbers of fungal gene copy numbers: 7.6, 6.5 and 6 (1og _10_) belonged to the FS treatment during Week 0, Week 3 and Week 6, respectively (Figure 3b). These results suggest that the fungus did not play an important role in the transformation of DNT.

### 3.3. Bacterial Community Diversity

**Figure 4:**
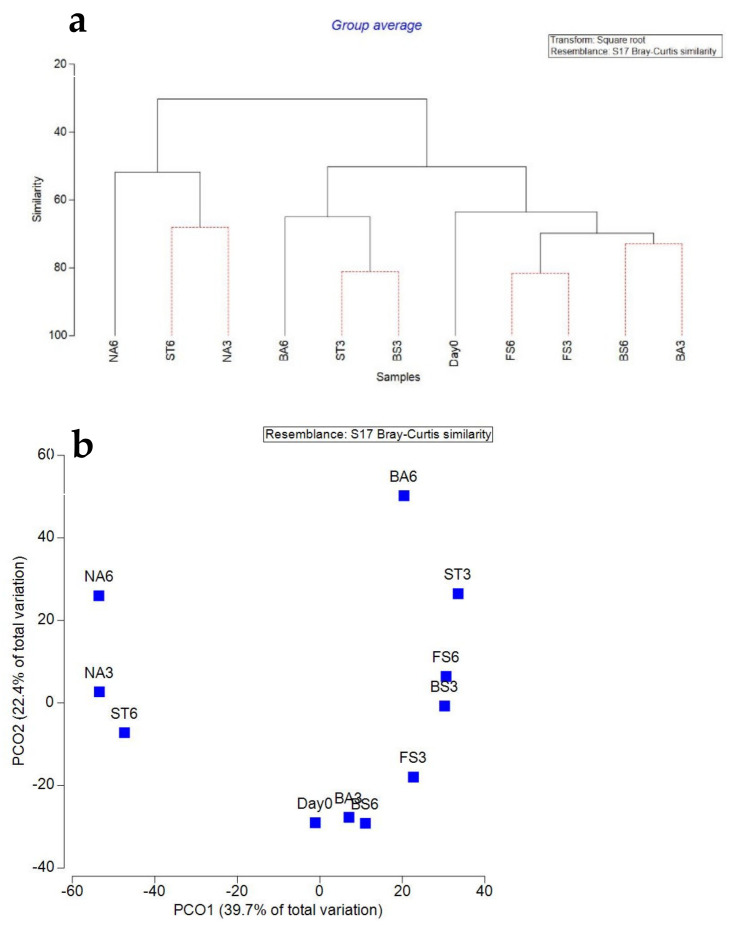
(**a**) UPGMA clustering analysis and (**b**) principal coordinate analysis (PCoA) constructs. NA: Natural attenuation; FS: *Fusarium solani*; BS: Biostimulation; BA: Bacilli, ST: *Streptomyces*; 3, 6: weeks.

16s rDNA sequencing was undertaken to further assess changes in the bacterial community during the transformation of DNT in the various treatments. The results showed a definitive shift in the microbial community in all treatments over the 6 weeks (Figure 4A). Cluster analysis based on UPGMA confirmed that inoculation or addition of P created an initial shift in bacterial communities at the start of the treatment when compared to the bacterial community present in the natural attenuation (control) samples (Figure 4A). In addition, principal coordinate analysis (PCoA) on Bray–Curtis matrices was performed to further assess the bacterial community differences between the treatments over the 6 weeks experiment (Figure 4B).

**Figure 5:**
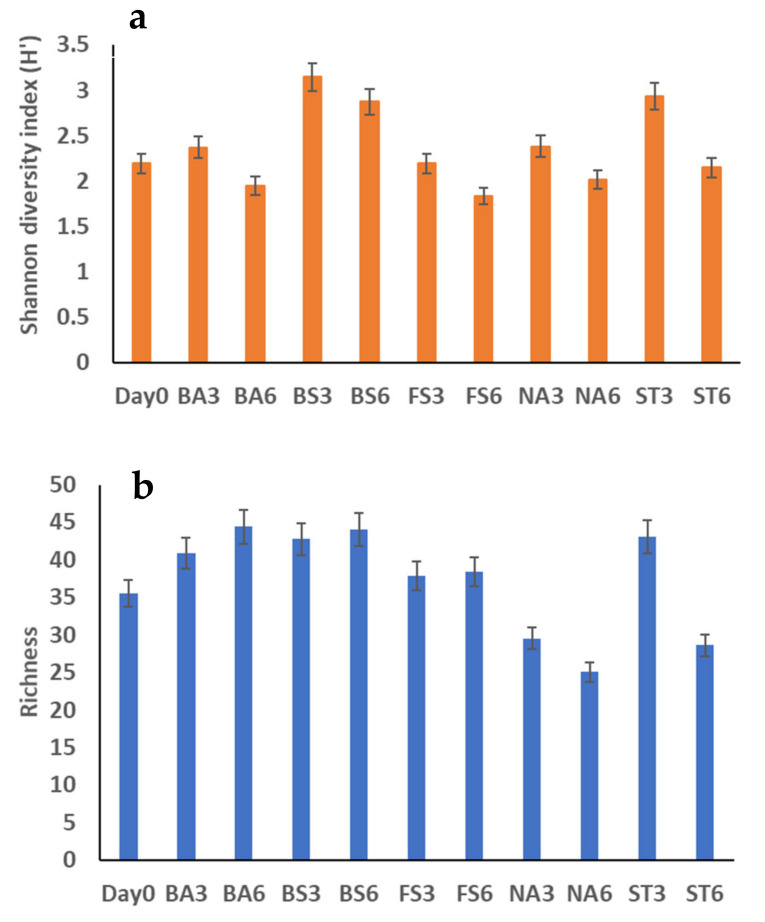
Bacterial diversity indices of samples for 6 weeks experiments. NA: Natural attenuation; FS: *Fusarium solani*; BS: Biostimulation; BA: Bacilli, ST: *Streptomyces*; 3, 6: weeks. (**a**) Shannon Diversity index (H’); (**b**) Richness.

PCoA is a visualisation method that shows the similarity or difference among the tested groups. Principal components 1 and 2 explained 34.66% and 33.71% of the total community variations, respectively (variance among groups). PCoA also indicated that the bacterial communities were different from Day 0 and from the natural attenuation control. The Shannon diversity index and richness values for the bacterial communities in all samples during the bioremediation generated from the MiSeq sequencing results are shown in Figure 5a,b. The results showed that the addition of P increased the Shannon diversity index in the biostimulation treatment; the highest Shannon diversity index, 3.14 was observed in the biostimulation treatment after 3 weeks; lowest values were observed in fungal addition treatments after 3 weeks, with an average of 1.84, confirming the different impacts of treatments on bacterial communities. In terms of richness, again, significant differences were observed among treatments. Biostimulation showed the richest community, with an average of 44, while natural attenuation showed the lowest values, with an average of 25 after 6 weeks.

**Figure 6:**
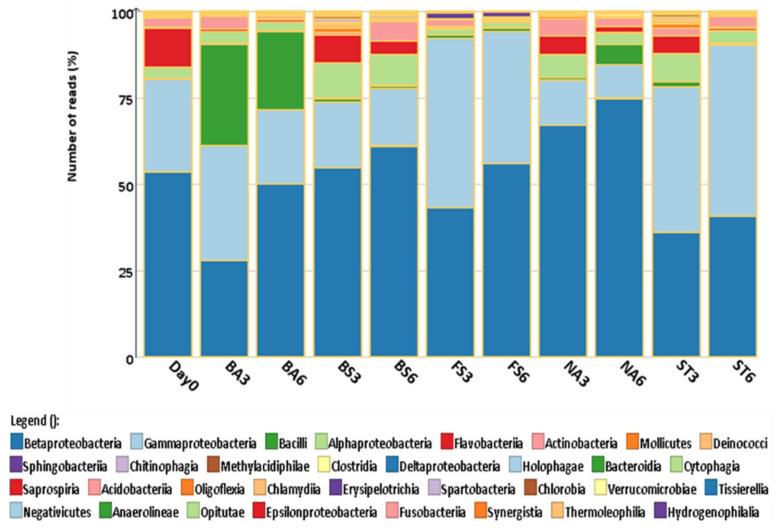
Variation in abundance of dominant bacterial classes at Day 0, week 3 and week 6 of different treatments assessed by 16S DNA sequencing analysis. NA: Natural attenuation; FS: *Fusarium solani*; BS: Biostimulation; BA: Bacilli, ST: *Streptomyces*; 3, 6: weeks.

Identification of the bacterial community at the class level present in the various treatments following the 6-week incubation in the microcosms is presented in Figure 6. The results showed that the community at Day 0 was dominated by Gammaproteobacteria, Alphaproteobacteria, Bacilli and Betaproteobacteria. However, a significant change in all treatments was observed by the end of the experiment. Not surprisingly, Bacilli remained the largest community in the Bacillus-amended treatment after 3 weeks’ incubation, but by the end of the experiment (6th week) the microbial community was dominated by Betaproteobacteria, suggesting a better adaptation to the contaminant by the indigenous microorganisms (Figure 6). This is consistent with the largest DNT transformation rates observed in the biostimulation treatment and their higher gene copy numbers compared to the control (Figure 3).

**Figure 7:**
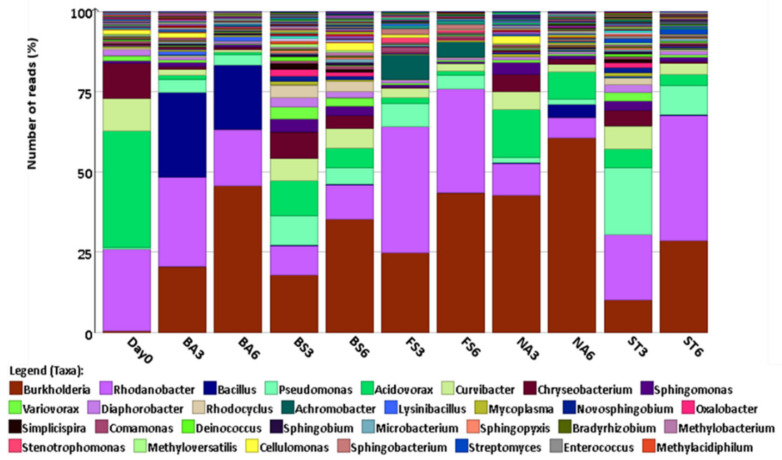
Bacterial diversity at genera level obtained from sequencing of 16S rDNA at week 6. NA: Natural attenuation; FS: *Fusarium solani*; BS: Biostimulation; BA: Bacilli, ST: *Streptomyces*; 3, 6: weeks.

Importantly, the microbial community in the biostimulation treatment remained relatively constant throughout the incubation with similarities to the bacterial community in the natural attenuation treatment (control), suggesting that the addition of nutrients created a more robust community, favouring the DNT degraders, as no additional C was added to the waste stream. Moreover, the genus *Burkholderia* was the dominant genus in the biostimulation treatment by the sixth week, followed by *Rhodanobacter*, *Acidovorax* and *Pseudomonas* (Figure 7). While *Pseudomonas* sp. have been previously reported as members of DNT-degrading consortia [3,4,24,36], several *Burkholderia* species have been shown to be capable of degrading DNT on their own and also as part of a consortia [4,13,37,38]. In fact, *Burkholderia* were the first isolated microorganisms capable of DNT degradation [39]. Thus, our results confirmed the presence of DNT-transforming microorganisms within the indigenous community. Therefore biostimulation (mainly by *Burkholderia* spp.) with nutrients resulted in the highest DNT transformation allowing the rapid growth and transformation of DNT.

## 4. Conclusions

The results showed that sludge containing DNT could be subjected to bioremediation. The results also showed that biostimulation resulted in the maximum transformation of DNT (from 300 to 55 g/L). Microbial assessment of the different treatments confirmed the presence of microorganisms capable of DNT transformation in the original sludge. However, the physicochemical condition of the original sludge, such as a lack of oxygen and P, prevented the activity of those microbes. Therefore, the addition of P with shaking led to the activation of the bacterial community involved in the transformation of DNT. Molecular analysis of the bacterial community suggested that the success of the bioremediation approach was founded on the minimal impact it had on the natural bacterial community while allowing for increased growth. This is especially important for a community that has been exposed to DNT for a long term, and their stimulation should be the first approach to bioremediate explosives-waste-contaminated sites.

## Figures and Tables

**Table 1:** Physical and chemical characteristics of the sludge used in this study.

Sludge Elements/Parameters	Units	Value
**pH**		9.2
**Organic C**	%	7.7
**Colour**		Yellow
**Nitrite**	mg/kg	1900
**Nitrate**	mg/kg	7200
**Sulphate**	mg/kg	6700
**Moisture**	%	41
**2,4,6 TNT**	mg/kg	<50
**2,4 DNT**	g/L	300
**2,6 DNT**	g/L	0.056
**RDX**		<50

**Table 2:** Description of the treatments used for the DNT bioremediation study.

Treatments	Symbol	Nutrient Formulation	Hydrocarbonoclastic Bacteria
**Natural attenuation (control)**	NA	-	None
**Bioaugmentation 1**	BA	-	Bacilli consortium
**Bioaugmentation 2**	ST	-	*Streptomyces* sp.
**Bioaugmentation 3**	FS	-	*Fusarium solani*
**Biostimulation**	BS	+	None
